# Small-angle Compton Scatter Artifact in Tc-99m-IDA Hepatobiliary Scintigraphy Resulting in the Breast Overlying the Liver in Planar Dynamic Imaging

**DOI:** 10.4274/mirt.galenos.2020.05658

**Published:** 2021-02-09

**Authors:** Mohsen Qutbi

**Affiliations:** 1 Shahid Beheshti University of Medical Sciences, Taleghani Educational Hospital, Department of Nuclear Medicine, Tehran, Iran

**Keywords:** Small-angle Compton scatter, artifact, liver, breast, hepatobiliary scan

## Abstract

Compton scatter photons are generally considered problematic in nuclear medicine imaging. Therefore, efforts are being made to minimize the involvement of these photons by employing some special strategies in daily practice. Basically, photons scattering at a small angle and traveling in the proper direction stand a chance of getting recorded and thereby participate in the image formation. These photons may create artifactual hot spots in the vicinity of a region with high concentration of radioactivity. The present study focuses on the negative impact of such photons during routine imaging in clinical setting, through an artifact detected in technetium-99m-IDA hepatobiliary scintigraphy, with the purpose of highlighting this issue to the nuclear medicine practitioners.

## Abstract (tr)

Compton saçılma fotonları genellikle nükleer tıp görüntülemesinde sorunlu olarak kabul edilir. Bu nedenle, günlük pratikte bazı özel stratejiler uygulayarak bu fotonların tutulumunu en aza indirmek için çaba gösterilmektedir. Temel olarak, küçük bir açıyla saçılan ve doğru yönde hareket eden fotonların kaydedilme ve dolayısıyla görüntü oluşumuna katılma şansı vardır. Bu fotonlar, yüksek radyoaktivite konsantrasyonuna sahip bir bölgenin çevresinde yapay sıcak noktalar oluşturabilir. Bu çalışma, bu konuyu nükleer tıp hekimlerine vurgulamak amacıyla, teknesyum-99m-IDA hepatobiliyer sintigrafisinde saptanan bir artefakt yoluyla, bu tür fotonların klinik ortamda rutin görüntüleme sırasında olumsuz etkisine odaklanmaktadır.

## Figures and Tables

**Figure 1:**
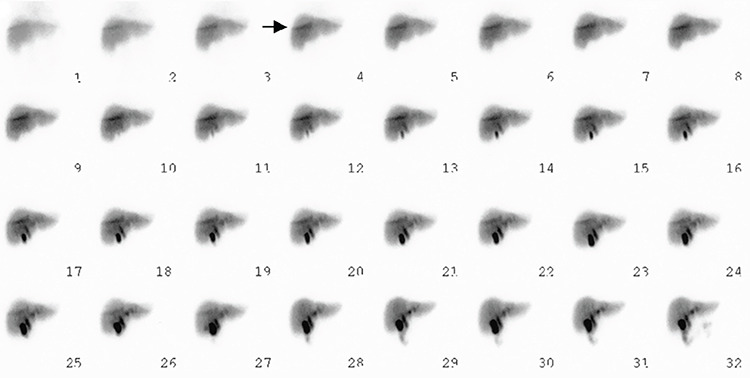
A 45-year-old woman was referred for a technetium-99m (Tc-99m)-mebrofenin hepatobiliary scan, which was performed in dynamic mode for 60 minutes using a single-head Genesys EPIC ADAC γ-camera with a general-purpose collimator and a window width of 20% for 140-Kev Tc-99m photons. The dynamic image showed attenuation of the right breast overlying the liver. Unexpectedly, an artifactual curvilinear zone of intense uptake (shown by arrow) was noted with an intensity higher than that in the unattenuated region of the liver, bordering attenuated and unattenuated regions and presumably coincident with the edge of the breast, from the beginning of the study. The visualization time and uptake pattern was not compatible with radiotracer excretion into the biliary system or any pathology in the liver. Later, in the dynamic phase (at frame 28 on the image), the breast was repositioned, and the curvilinear uptake subsequently disappeared. In practice, nuclear medicine images are formed mainly by photopeak with varying degrees of contribution of Compton scatter photons. The degree of this contribution depends on certain factors, such as width of the energy window. In Compton scattering, original photons emitted as a result of radioactive decay interact with the surrounding matter and consequently transfer a part of their energy to it. Thus, secondary photon travels in another direction with a lower energy as a function of the angle between the directions of the original and secondary photons (1,2). There are 2 mechanisms that can prevent recording of Compton scatter photons. The first mechanism is through physical collimation (i.e., using a lead collimator) that absorbs photons whose direction is not perpendicular to the collimator face or parallel to the axis of collimator holes. The second mechanism is accomplished by pulse height analyzer using an energy window to discard photons with energies that lie outside the desired range (1,2,3). Despite all these measures, photons that are scattered at a small angle, because of lower energy transfer, might be recorded. Practically, for a window width of 20%, only photons of Tc-99m reaching the camera detector in the range of 126-154 KeV meet the energy criteria to be accepted. Compton photons in proper direction with angle of scattering <53.5° also fulfill the energy criteria and are accepted by the system (1,4,5). In some circumstances, a higher proportion of Compton photons are produced, which was explained in an interesting experiment by Yeh (5). When air intervenes between regions of soft tissues, photons coming from one region scatter at a small angle to higher proportions when hitting the second region, thereby producing a false hot spot (5). One such phenomenon occurs along the lower edge of the breast, especially when it lies geometrically compared with the chest wall, in scans with high concentration of activity in the liver as in the present patient and produces a special artifactual pattern. Counts corresponding to the image in that region originate from the adjacent regions of the patient’s body. After repositioning of the breast, and thus change in the geometry of the breast over the chest wall, the false hot spot disappeared. Monte Carlo simulation is a useful technique to validate, although *in silico*, the formation of this artifact based on specific breast configuration and geometry on the chest and its elimination by simulated breast repositioning (6,7,8).
